# Supplementary material for: Predictors of Health-Workforce Job Satisfaction in Primary Care Settings: Insights from a Cross-Sectional Multi-Country Study in Eight African Countries
Source: Int J Environ Res Public Health. 2025 Jul 15;22(7):1108. doi: 10.3390/ijerph22071108 (PMC12295563; doi:10.3390/ijerph22071108)
Supplement: Supplementary file 1 [file ijerph-22-01108-s001.zip › ijerph-3576794-supplementary.pdf]

CS_02 SATISFACTION SURVEY FOR HEALTH WORKERS (INDICATOR 2.1)

Instructions

- This tool is to be administered to the community or facility health worker present in the health facility.
- Specific instructions for each question is in italics.
- Do not read out answer option but tick the most appropriate answer(s) based on the respondent's answer.
- Prior to asking the following questions explain to the client that they will use a ranking scale of 1-5 representing very dissatisfied to very satisfied, based on their perception and experience of the services they have utilized. Please mark their response in the appropriate column.
- Note down in much details as possible the responses to narrative questions.
- Inform that the questions are to be responded based on the past 12 months.
- Before you start the interview obtain written consent for the interview. Refer to the relevant consent sheet. Indicate if the health worker has agreed to the written consent:

☐ Health Worker has agreed *(continue the interview)*
☐ Health Worker has not agreed *(end the interview)*

(a) Country: <dropdown>

(b) District/County/Region: <dropdown>

(c) Health Facility Name: ____

(d) Health Facility Type: <dropdown>: Dispensary, Health Centre, Hospital, Health post (all other options from all country offices)

(e) Length of service: ___________ In this facility: _________

(f) Sex: ☐ Male ☐ Female

(g) Age: ________

(h) **Cadre:**

☐ Facility based: <Dropdown - Clinical Officers, Nurse, Midwife, Nurse and Midwife, Public Health Officers, Nurses, Lab technician, Medical Officer, Other> (If other, Specify: ______________)

☐ Community Based
If community based, the village they are working in: <Dropdown>

If community based:
☐ Paid salary ☐ Volunteering by being paid stipend ☐ Volunteering without stipend

#	Question	Not Applicable (NA)	1: Very Dissatisfied (VD)	2: Dissatisfied (D)	3: Neutral (N)	4: Satisfied (S)	5: Very Satisfied (VS)
**1.**	**EMPLOYEE-EMPLOYER RELATIONSHIP**						
How Satisfied are you with.......							
1.1	The support from the management of this institution						
1.2	Evaluation of your work based on a fair system of performance standards.						
1.3	The extent to which the rules of the institution makes it easy to do your job.						
1.4	If you have ranked "satisfied" or "very satisfied" for any of the above questions, please provide reasons:						
1.5	If you have ranked "dissatisfied" or "very dissatisfied" for any of the above questions, please provide reasons:						

#	Question	Not Applicable (NA)	1: Very Dissatisfied (VD)	2: Dissatisfied (D)	3: Neutral (N)	4: Satisfied (S)	5: Very Satisfied (VS)
**2**	**EMPLOYEE – EMPLOYER RELATIONSHIPS (CHWs ONLY including Volunteers)**						
2.1	Your working relationship with the village executive officer (or equivalent for the country)						
2.2	Communication regarding opportunities for the CHW						
2.3	CHW selection criteria and recruitment process process						
2.4	If you have ranked "satisfied" or "very satisfied" for any of the above questions, please provide reasons:						
2.5	If you have ranked "dissatisfied" or "very dissatisfied" for any of the above questions, please provide reasons:						
**3.**	**REMUNERATION & RECOGNITION**						
How Satisfied are you with…….							
3.1	Your pay being commensurate for the amount of work you do.<br><br>*For volunteers replace "pay" with "incentive"*						
3.2	Your pay being commensurate for your skills<br><br>*For volunteers replace "pay" with "incentive"*						
3.3	*Skip this question for volunteers*.<br><br>The bonuses and allowances you receive?						
3.4	Your efforts being recognized by the communities and other leaders						
3.5	If you have ranked "satisfied" or "very satisfied" for any of the above questions, please provide reasons:						
3.6	If you have ranked "dissatisfied" or "very dissatisfied" for any of the above questions, please provide reasons:						
**4.**	**PROFESSIONAL DEVELOPMENT**						
How Satisfied are you with…….							
4.1	The training and other opportunities to develop professionally or to develop your skills.						

#	Question	Not Applicable (NA)	1: Very Dissatisfied (VD)	2: Dissatisfied (D)	3: Neutral (N)	4: Satisfied (S)	5: Very Satisfied (VS)
4.2	The space and opportunity to learn new skills in this position						
4.3	The quality of blended trainings you have received (face to face and online training)						
4.4	If you have ranked “satisfied” or “very satisfied” for any of the above questions, please provide reasons:						
4.5	If you have ranked “dissatisfied” or “very dissatisfied” for any of the above questions, please provide reasons:						
4.6 [Ind 8.1]	Have you been trained to address issues in relation to climate change?<br><br>*If Yes, Then ask Question 4.6*	☐ Yes<br>☐ No					
4.7	If yes, what type of course was it and who was it provided by?	☐ Formal Training with certificate<br>☐ Informal Workshop or orientation<br>☐ Other (Specify): ___________<br><br>Training provider (institution):<br><br>_______________					
**5. PHYSICAL ENVIRONMENT AND FACILITIES**							
How Satisfied are you with…….							
5.1	The protection provided to you by the institution against occupational hazards (e.g.: Exposure to HIV)						
5.2	The sufficiency of equipment and material you are provided with to do your duties						
5.3	The safety of your physical environment to carry out your duties.						
5.4	*<u>This question is for Facility Based Health Workers only:</u>*<br><br>Housing facilities/housing allowance to facilitate your duties?						
5.5	If you have ranked “satisfied” or “very satisfied” for any of the above questions, please provide reasons:						

#	Question	Not Applicable (NA)	1: Very Dissatisfied (VD)	2: Dissatisfied (D)	3: Neutral (N)	4: Satisfied (S)	5: Very Satisfied (VS)
5.6	If you have ranked "dissatisfied" or "very dissatisfied" for any of the above questions, please provide reasons:						
**6**	**SUPPORTIVE SUPERVISION**						
6.1	How Satisfied are you with…….						
6.2	The amount of coaching and mentoring you receive from your direct supervisor						
6.3	The extent to which your suggestions are heard by your supervisor.						
6.4	The availability of your supervisor to answer work related questions						
6.5	If you have ranked "satisfied" or "very satisfied" for any of the above questions, please provide reasons:						
6.6	If you have ranked "dissatisfied" or "very dissatisfied" for any of the above questions, please provide reasons:						

7. Would you like to provide any other comment:

